# Bridging clinical insight and laboratory model in high-grade serous ovarian carcinoma (HGSOC) using DNA sequencing-based profiling of TP53

**DOI:** 10.18632/oncoscience.632

**Published:** 2025-10-14

**Authors:** Faisal Iqbal

**Affiliations:** ^1^Department of Pharmaceutical Sciences, University of Illinois Chicago, Chicago, IL 60612, USA

**Keywords:** HGSOC, OVCAR3, TP53

## Abstract

The most predominant and aggressive form of ovarian cancer is high grade serous ovarian carcinoma (HGSOC), characterized by late-stage diagnosis and poor prognosis. The TP53 gene, the molecular underpinnings of this malignancy studying *in vitro* model to serve as a valuable. The Sanger sequencing was used for clinical and laboratory wild type TP53 gene and making it an ideal profiling to offers a precise method for detecting comparable specific gene. In this study, drug repurposing agent’s metformin, chlorpromazine (CPZ) alone and combine were tested on both clinical and laboratory ovarian cancer samples to evaluate on hemocytometer and clonogenic assay for dead cell and proliferation respectively. Following drug treatment, both samples were further analyzed using Sanger sequencing to detect TP53 profiling. The resulting data were analyzed to achieve successfully known target region and worked as a bridge between clinical and laboratory model. The insights gained from this study not only validate OVCAR3 as a representative model for HGSOC but also provide a foundation for developing targeted therapeutic strategies.

## INTRODUCTION

The most prevalent and aggressive subtype of epithelial ovarian cancer is high grade serous ovarian carcinoma (HGSOC), approximately 70% of all ovarian cancer cases. HGSOC presents noteworthy challenges in treatment and prognosis to characterize by rapid development and late phase diagnosis. HGSOC is a complex molecular landscape involving a multitude of genetic alterations that drive tumorigenesis and therapeutic resistance [1–7].

The OVCAR3 cell line, serves as a valuable *in vitro* model for studying the molecular mechanisms underlying the disease while this cell line retains key genetic features of HGSOC, including TP53 and making it an ideal candidate for profiling studies. The Sanger sequencing method was used to enables the detection of specific DNA sequences, the methodology offer high sensitivity and accuracy, making it suitable for identifying targeted genes [8–13].

The OVCAR3 clinical and laboratory samples were used for drug repurposing to evaluate the data to implement the hemocytometer and clonogenic assay, while Sanger sequencing was used for TP53 gene profile and compared with both clinical and laboratory data. In conclusion, Sanger sequencing provides a powerful approach for genetic landscape of HGSOC that linked to tumorigenesis and therapeutic resistance, this unified methodology holds promise for the advancement of targeted therapies and personalized medication in the fight against high grade serous ovarian carcinoma (HGSOC) [14–19].

## RESULTS AND DISCUSSION

### OVCAR3 cell line of TP53 gene

The studying high-grade serous ovarian carcinoma (HGSOC), OVCAR3 cell line is a widely utilized *in vitro* model with advanced-stage ovarian cancer, OVCAR3 retains key molecular characteristics, including TP53 gene and makes it a precious tool for investigating the molecular mechanisms for HGSOC and assessing potential therapeutic approaches.

### Comparing drug repurposing of clinical and laboratory sample data

The hemocytometer was performed first time during cell suspension before 24 hours of using the drug, the second time after cell culture media changed, and third time after 24 hours of using the drug to check the drug’s cytotoxicity in the OVCAR3 cell line. The final volume in each 6 WP (well plate) was 2 ml.

The hemocytometer cell count data presented in the Table 1 provide insights into the responses of OVCAR3 cells to individual and combined drug treatments with metformin and CPZ.

**Table 1:** Hemocytometer based comparing drug repurposing of clinical and laboratory sample data

Sample	Drugs name	During cell suspension (Day 1)	After cell culture media changed (Day 2)	Day 4
Clinical	Control (No Drug)	0,1,1,0 (Answer: 2 × 10^4^)	0,0,1,2 (Answer: 3 × 10^4^)	0,2,1,0 (Answer: 3 × 10^4^)
–	Metformin (0.5 μM, 20 μl)	0,2,2,0 (Answer: 4 × 10^4^)	1,1,0,0 (Answer: 2 × 10^4^)	4,3,3,2 (Answer: 12 × 10^4^)
–	CPZ (2 μM, 20 μl)	3,0,0,1 (Answer: 4 × 10^4^)	1,0,1,2 (Answer: 4 × 10^4^)	2,2,7,3 (Answer:14 × 10^4^)
–	Combo (0.5 μM Metformin + 2 μM CPZ, 20 μl metformin + 20 μl CPZ)	1,2,0,0 (Answer: 3 × 10^4^)	2,1,1,0 (Answer: 4 × 10^4^)	7,3,3,3 (Answer: 16 × 10^4^)
Laboratory	Control (No Drug)	2,0,0,2 (Answer: 4 × 10^4^)	3,2,0,1 (Answer: 6 × 10^4^)	1,3,0,0 (Answer: 4 × 10^4^)
–	Metformin (0.5 μM, 20 μl)	3,1,0,1 (Answer: 5 × 10^4^)	1,1,1,1 (Answer: 4 × 10^4^)	7,3,3,5 (Answer: 18 × 10^4^)
–	CPZ (2 μM, 20 μl)	0,0,2,1 (Answer: 3 × 10^4^)	0,1,0,1 (Answer: 2 × 10^4^)	5,3,7,3 (Answer: 18 × 10^4^)
–	Combo (0.5 μM metformin + 2 μM CPZ, 20 μl metformin + 20 μl CPZ)	2,1,0,1 (Answer: 4 × 10^4^)	3,0,0,1 (Answer: 4 × 10^4^)	6,7,5,5 (Answer: 23 × 10^4^)

### Clinical sample drug repurposing data

In the first control group, the baseline dead cell count on day 1 was 2 × 10^4^, which increased modestly to 3 × 10^4^ after media change (day 2) and day 4 remain consistent 3 × 10^4^ dead cell counting and indicating without drug interference. Metformin at 0.5 μM initially had 4 × 10^4^ cells then dropping to 2 × 10^4^ after the media change, but then surging to 12 × 10^4^ after 24 hours of drug exposure, suggesting a delayed cell proliferation. Similarly, CPZ at 2 μM started at 4 × 10^4^, remained stable on day 2 and increased dead cells to 14 × 10^4^ on day 4 after 24 hours of drug treatment. Interestingly, the combination treatment (0.5 μM metformin + 2 μM CPZ) started with 3 × 10^4^ dead cells, next day increased slightly to 4 × 10^4^ and then showed dead cell to 16 × 10^4^ by day 4. The Table 1 data indicated that at low concentrations both drugs alone and combined may have even triggered proliferation and only few dead cell were counted.

### Laboratory sample drug repurposing data

**Figure 1:**
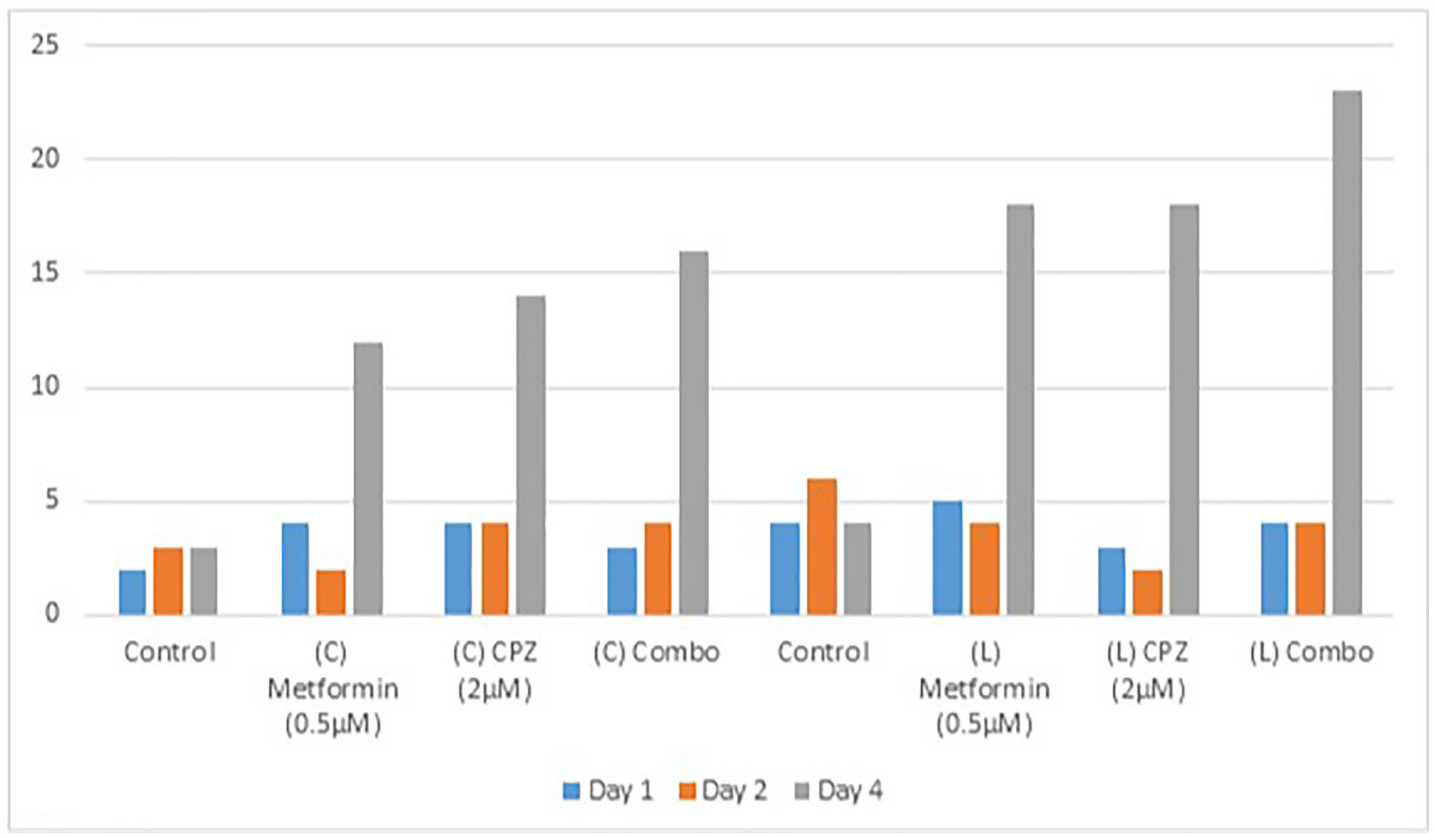
Hemocytometer dead cells count. Here is the bar graph showing the hemocytometer based dead cell counts for each treatment group across day 1, day 2 and day 4 (24 hours post treatment). The data clearly indicated that dead cells increased in combo with high concentration drug group. The C and L is presenting clinical and laboratory samples respectively and each group has separate control.

In the Table 1 laboratory control group, dead cells increased steadily from 4 × 10^4^ on day 1, day 2 was 6 × 10^4^ and day 4 was drop to 4 × 10^4^. Metformin at 0.5 μM started at 5 × 10^4^, remained stable (4 × 10^4^) on day 2, and sharply increased dead cells to 18 × 10^4^ on day 4, again indicating drug exposure. CPZ at 2 μM began at 3 × 10, dropped to 2 × 10 then 18 × 10. Notably, the combo treatment (0.5 μM metformin + 2 μM CPZ) had 4 × 10 cells on day 1 and day 2, which then rose to 23 × 10 on day 4, the highest dead cells observed in this group. These results suggest that drug repurposing did not suppress cells proliferation but might have triggered adaptive responses in OVCAR3 cells (Figure 1).

### Clonogenic assay comparing clinical and laboratory sample data of OVCAR3 cell line

After drug treatment, the clonogenic assay was used for colonies counting to evaluate the data.

The clonogenic assay clinical sample results presented in the Table 2, evaluate the cytotoxic effects of Metformin, CPZ and their combination on OVCAR3 cells. The first control yielded 90 colonies, representing a 90% plating efficiency (PE). Treatment with metformin at 0.5 μM (20 μl) reduced the colony count to 77, resulting in a surviving fraction (SF) of 85.55%, while CPZ at 2 μM (20 μl) produced 75 colonies with an SF of 83.33%. The combination of metformin and CPZ further reduced the colonies to 60, corresponding to a significantly lower SF of 66.66%.

**Table 2:** Clonogenic assays comparing clinical and laboratory sample data

Sample	Condition	Colonies	Control colonies	PE%	SF%
Clinical	Control	90	90	90	
–	Metformin (0.5 μM)	77	90		85.55
–	CPZ (2 μM)	75	90		83.33
–	Combo (0.5 μM + 2 μM)	60	90		66.66
Laboratory	Control	92	92	92	
–	Metformin (0.5 μM)	73	92		79.35
–	CPZ (2 μM)	71	92		77.17
–	Combo (0.5 μM + 2 μM)	55	92		59.78

**Figure 2:**
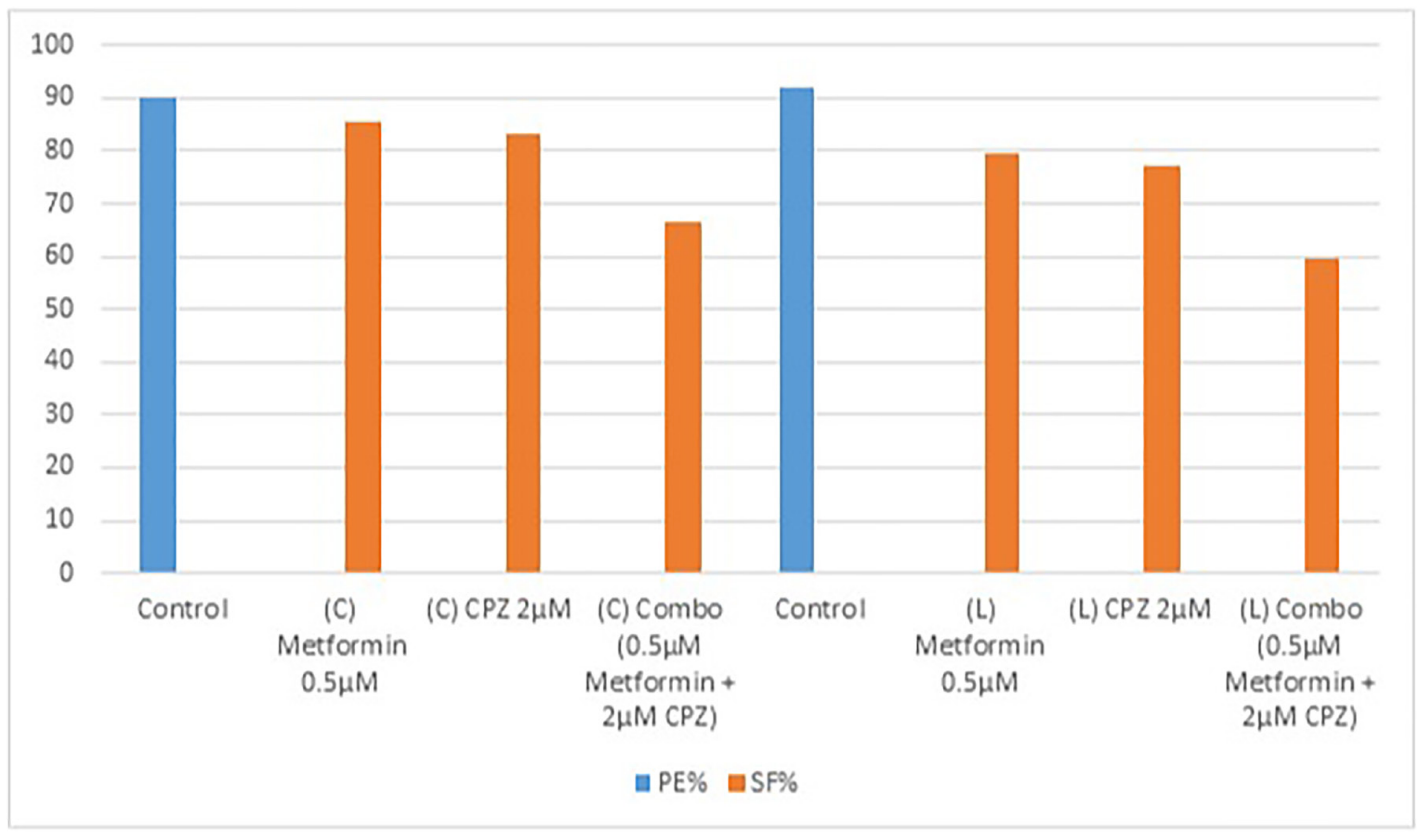
Clonogenic assay bar graph. The data demonstrated that CPZ and metformin alone reduce the formation of colonies while combo was more effective. The blue bar graph are presenting plating efficiency (PE) of untreated control of each group (clinical and laboratory) and orange bar graph presenting surviving fraction (SF).

In Table 2 laboratory sample, the control produced 92 colonies (PE = 92%), while metformin at 0.5 μM (20 μl) and CPZ at 2 μM (20 μl) reduced colony numbers to 73 and 71, resulting in SF values of 79.35% and 77.17% respectively. The combination treatment (0.5 μM metformin + 2 μM CPZ, 20 μl each) further decreased colony formation to 55 colonies, corresponding to SF of 59.78% (Figure 2).

### Sanger sequencing-based profiling of TP53

In this study, Sanger sequencing was employed to characterize TP53 status in OVCAR3 cell line, a widely used *in vitro* model for HGSOC. The successful sequencing was performed to compare the wild type clinical sample with wild type laboratory sample. The Sanger sequencing remain gold standard method and used for known target sequence with high precision and base calling quality are needed. The sequencing chromatogram of both wild type clinical and wild type laboratory samples were obtained from reverse primer and confirmed the accuracy of the data. The chromatograms base calling quality was fast and clear with minimal noise in background. The sequencing alignment reads the TP53 which was well characterized the HGSOC. The findings were consistent and validated the methodology. Sanger sequencing remain desirable for targeted low throughput analysis with high base precision. It is highly suitable when confirming the wild type target sequence with limited number of sample. It is simple, cost effective and less use of bioinformatics requirements and make it approachable for most molecular laboratories. So the Sanger sequencing application for TP53 profiling provided reliable and high quality data and confirmed the comparable wild type. These outputs not only validated the genetic profile of OVCAR3 cell line but also helpful in ovarian cancer research.

### Clinical implication

**Figure 3:**
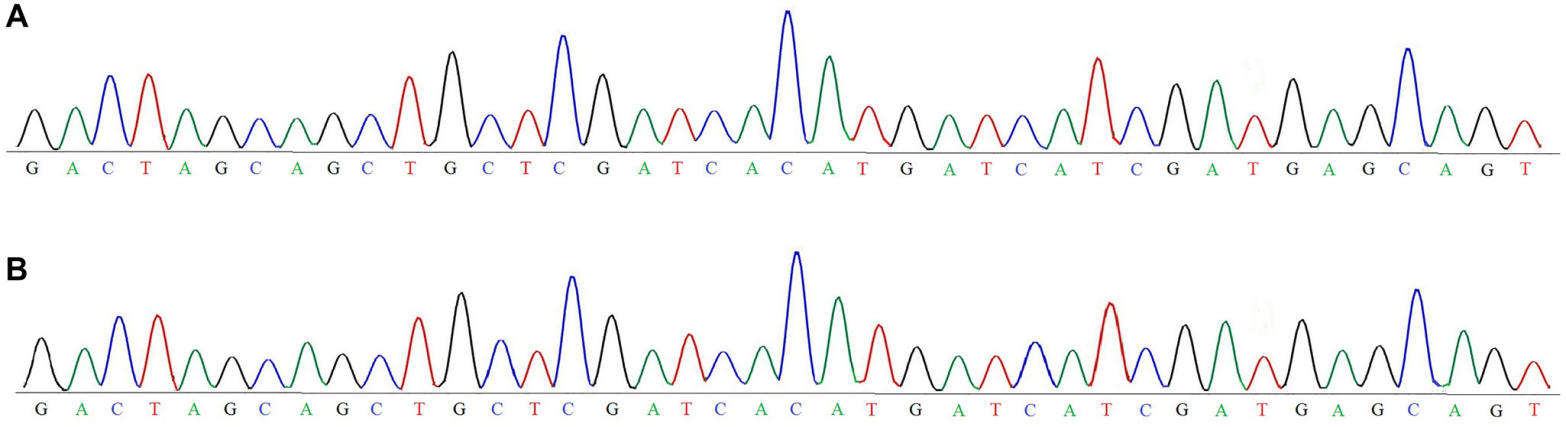
Sanger sequencing for TP53 gene profiling. Sample (**A**), derived from a clinical while sample (**B**) is a laboratory based ovarian cancer, both similar known target region was used for sequencing. Sanger sequencing showed precise and high quality results, the chromatograms confirmed the presence of wild-type TP53 sequence in both cases, indicating no mutations in the targeted region. These findings not only verify the genetic consistency and authenticity of the OVCAR3 model but also support its relevance as a preclinical tool in ovarian cancer research. The use of Sanger sequencing provided accurate base level resolution and comparative molecular analysis across clinical and experimental settings.

The OVCAR3 cell line identification provide opportunities for targeted therapeutic intrusions, though comprehensive molecular profiling to guide personalized treatment approaches. This study engaged Sanger sequencing to investigate the OVCAR3 cell line landscape for high grade serous ovarian carcinoma (HGSOC). The Figure 3 findings align with existing literature and underscoring the utility of OVCAR3 in bridging laboratory models with clinical insights. The Sanger sequencing analysis identified a TP53 gene in OVCAR3 cell line, a trademark of HGSOC. The use of Sanger sequencing allowed for fast and quantitative detection of nucleotides within a precise target region of TP53. This technique offers numerous advantages including higher sensitivity for detecting low-frequency variants. Particularly, Sanger sequencing is highly suitable for genetic detection and information that can be helpful for understanding the tumor.

The current study demonstrate the value of OVCAR3 as an appropriate *in vitro* model for TP53 oncogene and drug resistance in HGSOC. The integration of molecular approach such as Sanger sequencing into preclinical workflows strengthens the relevance of cell-based assays and ensures that genetic context is correctly considered during drug screening. The recent study suggest that the efficacy of certain drug combinations including repurposing agents like chlorpromazine (CPZ) and metformin may vary significantly depending on TP53 status. In conclusion, Sanger sequencing based analysis confirmed the comparable clinical and laboratory wild type TP53 gene, this approach offers a fast and consistent technique for gene profiling and can be adapted for broader use in ovarian cancer research and diagnostics.

## MATERIALS AND METHODS

### OVCAR3 cell line

The OVCAR3 cell line (ATCC) was cultured in RPMI-1640 medium supplemented with fetal bovine serum (FBS) 10% and penicillin-streptomycin 1%. At 37°C cells were sustained with 5% CO_2_.

### Hemocytometer

A manual cell counting device call hemocytometer was commonly used to define the cell feasibility especially dead cells. In this study culture OVCAR3 cells were harvested, trypsinization for adherent cells and resuspended for suitable volume of PBS or culture medium. A 1:1 ratio mixture of the cell suspension with 0.4% trypan blue dye was prepared to differentiate between live and dead cells. Live cells reject the dye and appear clear while dead cells take up the dye and appear blue. A cell suspension stained was loaded 10 μl on hemocytometer and grid define the known volume. The chamber filled the cell suspension with capillary action then cells were counted under microscope in specific square. Only cells within the square and those touching the upper and left borders are counted to avoid duplication. The number of cells per ml is calculated by multiplying the average cell count per square by the dilution factor and a standard conversion factor (10). Evaluation of cell feasibility is dividing the number of live cells by the total number of cells and multiplying by 100 to get a percentage. This protocol provides a reliable, low-cost method for quantifying both total and viable cells in a given population. The purpose of hemocytometer used was to count the dead cell and check the impact of drugs that creating resistance or cytotoxicity.

### Clonogenic assay

A gold standard method clonogenic assays were used for the evaluation of OVCAR3 ovarian cancer cell line for proliferative and survival of cancer cells after exposure of drug treatment. In this study, OVCAR3 cells were first cultured under standard conditions in RPMI-1640 medium supplemented with 10% fetal bovine serum and 1% penicillin-streptomycin. Once the cells reach exponential growth, trypsinize and counted using a hemocytometer with different time frames in different conditions and seeded at low densities in 6-well plates to ensure that colonies arise from single cells. After allowing the cells to adhere overnight, changed the media and next day cell were treated with the desired drugs CPZ, metformin and combo compare with control (no drug) for a defined duration (commonly 24–72 hours) according to the cells condition. During this period, 50 or more cells were considered per colony. At the end of the incubation period, the medium was gently removed and cells were fixed with methanol and stained with 0.5% crystal violet for 15 minutes. Distilled water was used for excess stain to wash off and plates are airdried. Colonies were counted manually on fluorescent microscope. The plating efficiency (PE) and surviving fraction (SF) were calculated to evaluate the treatment of cytotoxic effect. PE is the percentage of seeded cells that form colonies in untreated controls while SF is the ratio of colonies formed after treatment to the number of cells seeded, normalized to PE. This assay provides a sensitive measure of a treatment’s ability to inhibit reproductive viability in cancer cells over time.

### DNA extraction

OVCAR3 cell line of TP53 gene, clinical and laboratory sample DNA was extracted to perform the application of PCR and Sanger sequencing. The combo of both samples cell plate was used to extract the DNA. First culture the OVCAR3 cells in RPMI-1640 medium supplemented with fetal bovine 10% and 1% penicillin-streptomycin at 37°C for 5% CO_2_. When the cell confluence reach 80%, the medium was aspirated and washed the cells with 1 ml of sterile PBS. Cells were detached using 0.25% trypsin-EDTA and neutralized with fresh medium. The suspension was transferred in 15 ml centrifuge tube and spun at 1000 × g for 2 minutes to pellet the cells. The supernatant was discarded and the pellet was resuspended in 200 μl of PBS. DNA was then extracted using a silica column-based kit such as the Qiagen DNeasy Blood and Tissue Kit. The resuspended cell pellet was lysed by adding 20 μl of Proteinase K and 200 μl of Buffer AL, followed by incubation at 55°C for 10 minutes to ensure complete cell lysis. After lysis, 200 μl of 100% ethanol was added to precipitate DNA and the mixture is transferred to a spin column. The column was centrifuged and the bound DNA was washed with Buffer AW1 and AW2 to remove proteins and salts. Finally, DNA was eluted in 50 μl of AE buffer or nuclease-free water.

The purity and concentration of the DNA were evaluated on NanoDrop.

### Primer design

Target genes commonly in HGSOC for clinical and laboratory sample, including TP53 were selected for gene profiling. Primers were synthesized by Integrated DNA Technologies (IDT, USA). Forward primer (CCCATGGCATCCTAGTGAAA) and reverse primer (CAAAGGTCCGGAAGTTGTGG). While reverse primer was also used for Sanger sequencing.

### PCR reaction setup for Sanger sequencing

Polymerase chain reaction (PCR) was performed to generate DNA template for Sanger sequencing, total reaction volume was 25 μl for each sample. The reaction mixture contain 10 ng of DNA template, dNTP mix (10 mM each) 0.5 μl, 10X PCR buffer 2.5 μl, 25 mM MgCl_2_ for 1.5 μl, 0.5 μl each of forward and reverse primers (10 μM), Taq DNA polymerase (5 U/μL) 0.25 μl and nuclease-free water to complete the final volume. Thermal cycling was carried out under the following conditions, the initial denaturation at 94°C for 3 minutes with followed by 30 amplification cycles of denaturation at 94°C for 30 seconds, primer annealing at 54°C for 30 seconds and extension at 72°C for 30 seconds with a final extension step at 72°C for 5 minutes. PCR products were then purified to remove residual primers, nucleotides, enzymes and buffer components using a silica column-based purification kit (QIAquick PCR Purification Kit, Qiagen). The purified amplicons were quantified and approximately 10 μl of PCR product (probably 10 ng/μl) was submitted for Sanger sequencing along with 5 μl of sequencing primer (5 μM), ensuring that only one primer was used per reaction.

### Sanger sequencing of TP53 in OVCAR3 cells

The sequencing reactions were performed using an automated capillary electrophoresis system (e.g., Applied Biosystems 3500) and BigDye^™^ Terminator v3.1 Cycle Sequencing Kit. Raw sequencing chromatograms were analyzed on software.

## CONCLUSIONS

In conclusion, these findings enhance our understanding of the cellular and molecular mechanisms driving HGSOC and inform the development of targeted therapies. The OVCAR3 model continues to be instrumental in bridging laboratory research and clinical applications, offering valuable insights into the complexities of ovarian cancer.
